# Research and development of an electron beam focusing system for a high-brightness X-ray generator

**DOI:** 10.1107/S0909049510029948

**Published:** 2010-11-05

**Authors:** Takeshi Sakai, Satoshi Ohsawa, Noriyoshi Sakabe, Takashi Sugimura, Mitsuo Ikeda

**Affiliations:** a Accelerator Laboratory, High Energy Accelerator Research Organization (KEK), 1-1 Oho, Tsukuba, Ibaraki 305-0801, Japan; b Photon Factory, High Energy Accelerator Research Organization (KEK), 1-1 Oho, Tsukuba, Ibaraki 305-0801, Japan; c Foundation for Advancement of International Science, 586-9 Akatsuka, Tsukuba, Ibaraki 305-0062, Japan

**Keywords:** X-ray generator, high-flux electron beam, DC electron gun, combined-function bending magnet

## Abstract

A new type of rotating anticathode X-ray generator, where an electron beam of up to 60 keV irradiates the inner surface of a U-shaped Cu anticathode, has achieved a beam brilliance of 130 kW mm^−2^ (at 2.3 kW). A higher-flux electron beam is expected from simulation by optimizing the geometry of a combined-function-type magnet instead of the fringing field of the bending magnet. In order to minimize the size of the X-ray source the electron beam has been focused over a short distance by a new combined-function bending magnet, whose geometrical shape was determined by simulation using the *Opera-3D*, *General Particle Tracer* and *CST-STUDIO* codes. The result of the simulation clearly shows that the role of combined functions in both the bending and the steering magnets is important for focusing the beam to a small size. FWHM sizes of the beam are predicted by simulation to be 0.45 mm (horizontal) and 0.05 mm (vertical) for a 120 keV/75 mA beam, of which the effective brilliance is about 500 kW mm^−2^ on the supposition of a two-dimensional Gaussian distribution. High-power tests have begun using a high-voltage 120 kV/75 mA power supply for the X-ray generator instead of 60 kV/100 mA. The beam focus size on the target will be verified in the experiments.

## Abstract 2

In order to minimize the size of the X-ray source for a U-shaped rotating anticathode X-ray generator, the electron beam is focused over a short distance by a combined-function bending magnet. Simulation predicts that the beam brightness will reach almost 500 kW mm^−2^ for a 120 keV/75 mA beam.

## 1. Introduction

Beam power and beam size are important for high-brightness X-ray generation. However, conventional rotating anti­cathode X-ray generators have severe operational limits such as the beam power density not exceeding the melting point of the irradiated target as well as electric discharge and surface damage of the target caused by thermal stress. These factors are obstacles to increasing the X-ray intensity. However, a U-shaped rotating anticathode was developed for X-ray generators in order to overcome such limitations (Sakabe, 1995 ▶; Sakabe *et al.*, 2008 ▶). For the electron gun for this generator, a thermionic type was introduced with an aperture grid electrode instead of a conventional mesh grid. In addition, a beam focusing system was adopted using a magnetic lens, a quadrupole and a 180° bending magnet (Ohsawa, 2004 ▶, 2005 ▶, 2008 ▶; Sugimura *et al.*, 2007 ▶, 2008 ▶). A beam brilliance of 130 kW mm^−2^ (at 2.3 kW) has been achieved to date in experiments using the new X-ray generator with the U-shaped rotating anticathode (Ohsawa *et al.*, 2008 ▶).

Improvements to the system are still under way. We have raised the new target value of the beam brilliance to 300 kW mm^−2^. An increase in the beam brightness is expected from beam simulations if we improve the focusing and bending magnets system (Sakai *et al.*, 2009 ▶).

## 2. U-shaped Cu anticathode for the X-ray generator

### 2.1. X-ray generator overview

**Figure 1:**
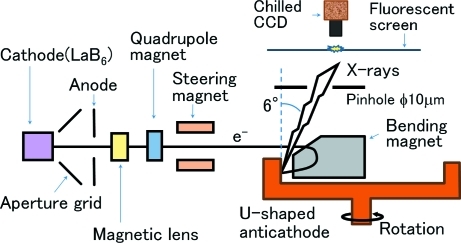
Schematic view of the new X-ray generator with the U-shaped rotating anticathode. The X-ray generator is comprised of three sections: electron gun, beam focus system and 180° bending magnet.

Figs. 1 ▶ and 2 ▶ show, respectively, a schematic view and a photograph of the X-ray generator with the U-shaped rotating anticathode. The X-ray generator is comprised of three sections: electron gun, beam focus system and 180° bending magnet. The electron beam irradiates the inner surface of the U-shaped rotating anticathode (see Fig. 1 ▶). The electron gun and the bending magnet are housed within a vacuum chamber. The X-ray take-off angle is 6° to the surface of the anticathode so as to compress the elliptic-shaped electron focus spot in the rotating-axis direction and makes it a circle when being seen as an X-ray source.

### 2.2. Electron gun section

The electrode geometry of the electron gun was optimized, based on EGUN (Herrmansfeldt, 1988 ▶) simulation results (Sugimura *et al.*, 2007 ▶, 2008 ▶). Instead of a conventional mesh grid electrode, an aperture grid electrode was adopted in consideration of the emittance and grid heating. The cathode is made from LaB_6_ and is 2 mm in diameter. The operating grid potential of the electron gun has been optimized to be 3 kV higher than the cathode potential.

### 2.3. Beam focusing section

**Figure 3:**
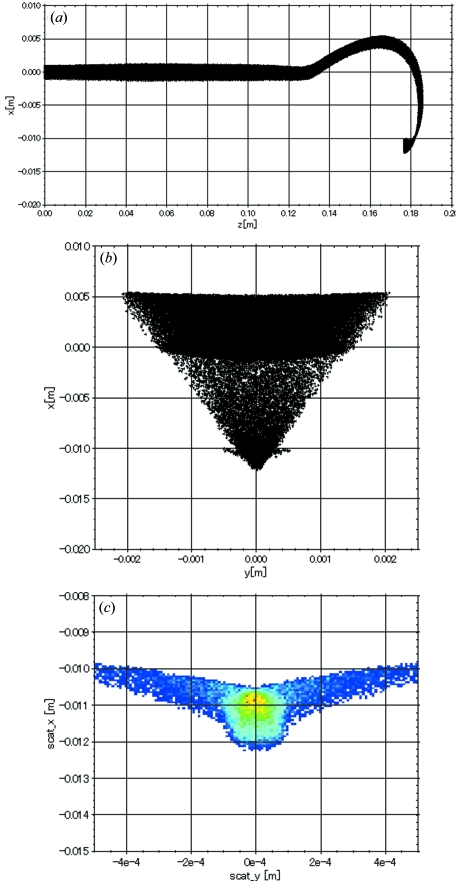
Beam trajectories were simulated from the electron gun to the anticathode target using the *GPT* code for a beam of 60 keV/38 mA. (*a*) Side view of the beam trajectory. (*b*) Front view of the beam trajectory. (*c*) Beam profile on the target.

The beam focusing section is composed of a magnetic lens, a quadrupole magnet and a steering magnet. The magnetic lens and Q-magnet are introduced to obtain small beam sizes on the target in both the horizontal and vertical directions. The steering magnet is used to optimize the horizontal incidence angle into the bending magnet, as shown in Fig. 3(*a*) ▶, in which the horizontal direction is shown vertically.

### 2.4. Beam bending magnet section

The electron beam is focused strongly over a short distance by the bending magnet as well as being bent by it. This is due to the effects of a fringing field in the incidence plane of the 180° bending magnet (see Fig. 1 ▶).

Table 1 ▶ summarizes the specifications of the new X-ray generator. The maximum power is 120 kV and 75 mA; however, since high-power tests have just begun, the tested power ranges covered are up to 60 kV/100 mA and 120 kV/10 mA.

## 3. Simulation and verification by experiment with beam

### 3.1. Magnet simulation and beam simulation

**Figure 4:**
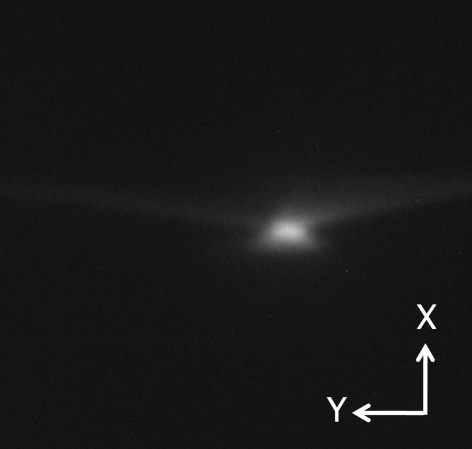
Experimental example of a beam profile on the anticathode target (60 keV/38 mA beam).

The bending-magnet field was firstly calculated using the code *Opera-3D* (Vector Fields; http://www.vectorfields.com/). Then the beam trajectories were simulated from the electron gun to the anticathode target with the code *General Particle Tracer* (*GPT*) (Pulsar Physics; http://www.pulsar.nl/) using the magnetic field data previously obtained with *Opera-3D*. Fig. 3 ▶ shows a typical simulation result. The beam trajectories [Figs. 3(*a*) and 3(*b*) ▶] and the beam spot shape on the target (Fig. 3*c* ▶) were calculated using the space-charge effect for a beam of 60 kV/38 mA. The simulation result is consistent with an experiment performed under the same beam conditions (Fig. 4 ▶).

### 3.2. Problems and solutions of the bending magnet

In §2.4 the fringing magnetic field at the cut corner was used for focusing the beam in the 180° bending magnet, for which the pole pieces were parallel to each other. Therefore, as seen in Fig. 3(*b*) ▶, the beam does not experience focusing forces inside the bending magnet. Furthermore, the beam spot on the target is partly out of focus, as shown in Fig. 3(*c*) ▶. It is obvious that the aberration of the magnet is not negligible in finer focusing.

For these reasons the bending magnet has been improved to a combined-function type, in which the magnetic pole faces are sloped so that the beam always experiences a focusing force from the magnetic field as well as a bending force. In addition, it has turned out to be effective for fine focusing to optimize the pole shape of the steering magnet which is in front of the bending magnet. On the other hand, there are other possibilities for improvement. For example, the simulation predicts more suitable distances for each magnet; we have not yet adjusted these distances.

### 3.3. New magnet simulation

**Figure 5:**
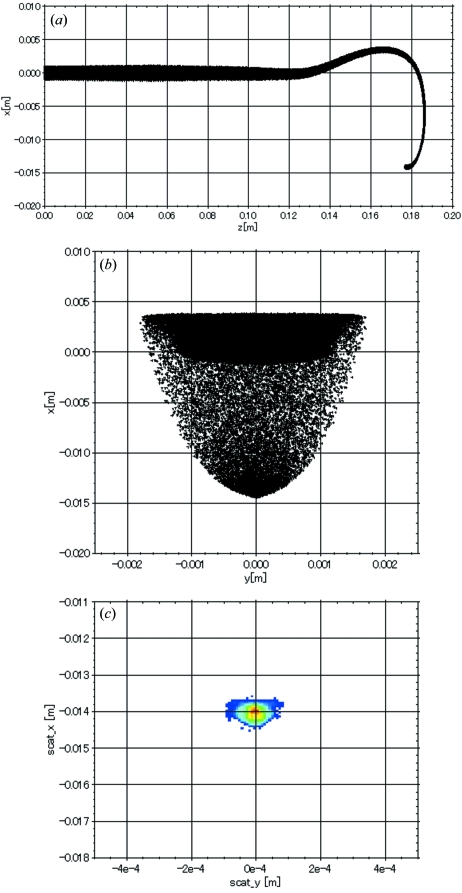
Simulation results of the new magnet system for a 120 keV/75 mA beam. (*a*) Side view of the beam trajectory. (*b*) Front view of the beam trajectory. (*c*) Beam profile on the target. FWHM sizes of the beam are predicted to be 0.45 mm (*x*: horizontal) and 0.05 mm (*y*: vertical) for which the effective brilliance is about 500 kW mm^−2^ with the supposition of a two-dimensional Gaussian distribution.

For the new magnet system the beam trajectories were simulated using the *Opera-3D* and *GPT* codes as previously (§3.1). Fig. 5 ▶ shows the simulation results for a beam of 120 keV/75 mA. It can be seen from the simulation results in Fig. 5(*b*) ▶ that the beam is experiencing the focusing force until just before the anticathode target. FWHM sizes of the beam are predicted in Fig. 5(*c*) ▶ to be 0.45 mm (*x*: horizontal) and 0.05 mm (*y*: vertical), for which the effective brilliance is about 500 kW mm^−2^ with the supposition of a two-dimensional Gaussian distribution.

### 3.4. Verification experiment

**Figure 6:**
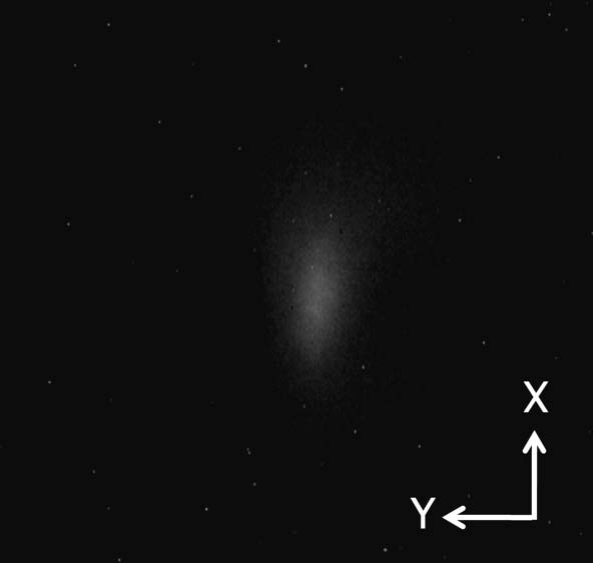
A typical example of an experimental beam profile on the anticathode target observed for a 120 keV/10 mA beam. The beam aberration became obviously smaller than before, observed in the previous system by use of the fringing field.

After modifying the bending magnet to a combined-function type, we carried out beam tests in the 60–120 kV region, but the current was limited to up to 10 mA. As can be seen in the beam profile for the 120 keV/10 mA beam and an aperture grid voltage of 1.6 kV, shown in Fig. 6 ▶, the beam aberration became obviously smaller than before observed at 60 kV. We can see from Fig. 5 ▶ that the bending magnet has the expected function for horizontal beam focusing. However, the vertical size is large. We optimized the system at 120 keV/75 mA. Therefore it is necessary to carry out tests in the high-power region to confirm this performance. We have recently obtained a 120 kV power supply and have just begun beam tests in the higher beam current region.

### 3.5. Remaining issues and challenges for the future

**Figure 7:**
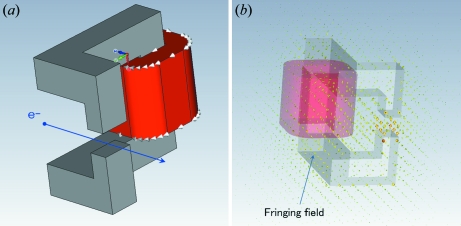
(*a*) Schematic view of the steering magnet. The present steering magnet has a complicated shape owing to limited installation space. (*b*) Calculated example using *CST-STUDIO* showing clearly that the fringing field is not negligibly small. The beam is easily influenced by the field.

The simulations and experiments revealed some issues about the focusing and bending magnets. The positions and the geometric field shapes have not yet been optimized for all of the magnets. In particular, the present steering magnet has a complicated shape owing to the limited installation space, as can be seen in Figs. 7 ▶ and 8 ▶. The distance from the Q-magnet to the bending magnet is relatively large. Therefore, if the fringing field is not negligibly small on the beamline the electron beam will be easily influenced by the field.

**Figure 8:**
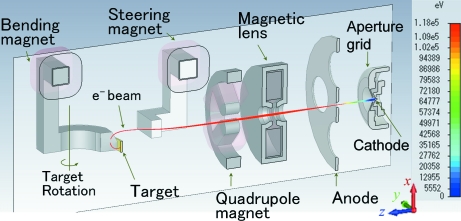
Vertical cross section of the computation model in *CST-STUDIO*. The entire realistic beam trajectory from the gun to the target is shown in red for a 120 keV/75 mA beam with an aperture grid voltage of 3 kV.

A more realistic simulation is required for detailed studies. The *CST-STUDIO* code (http://www.cst.com/) makes it easy to simulate the more realistic beam trajectory including the fringing field. Fig. 8 ▶ shows the computation model in *CST-STUDIO*. The new simulation made it clear that the fringing field of the steering magnet was not negligibly small, and it should be decreased by using magnetic shielding or simplifying its shape (for instance, C-type). It is expected that the new code will be more useful for improving the beam focusing by simulating the entire beam trajectories from the gun to the target. Further optimization will be necessary.

## 4. Conclusion

The results of the simulations and the experiments clearly show that the beam brightness is expected to increase with the decrease of aberration owing to the improvement of the pole faces of the bending and steering magnets. Simulation shows that the beam brightness will reach about 500 kW mm^−2^ for a 120 keV/75 mA beam. However, optimization in real systems is still insufficient, and further realistic simulations of the beamline will be necessary. It is expected that the beam brightness will increase further by optimizing the entire beamline.

## Figures and Tables

**Figure 2:**
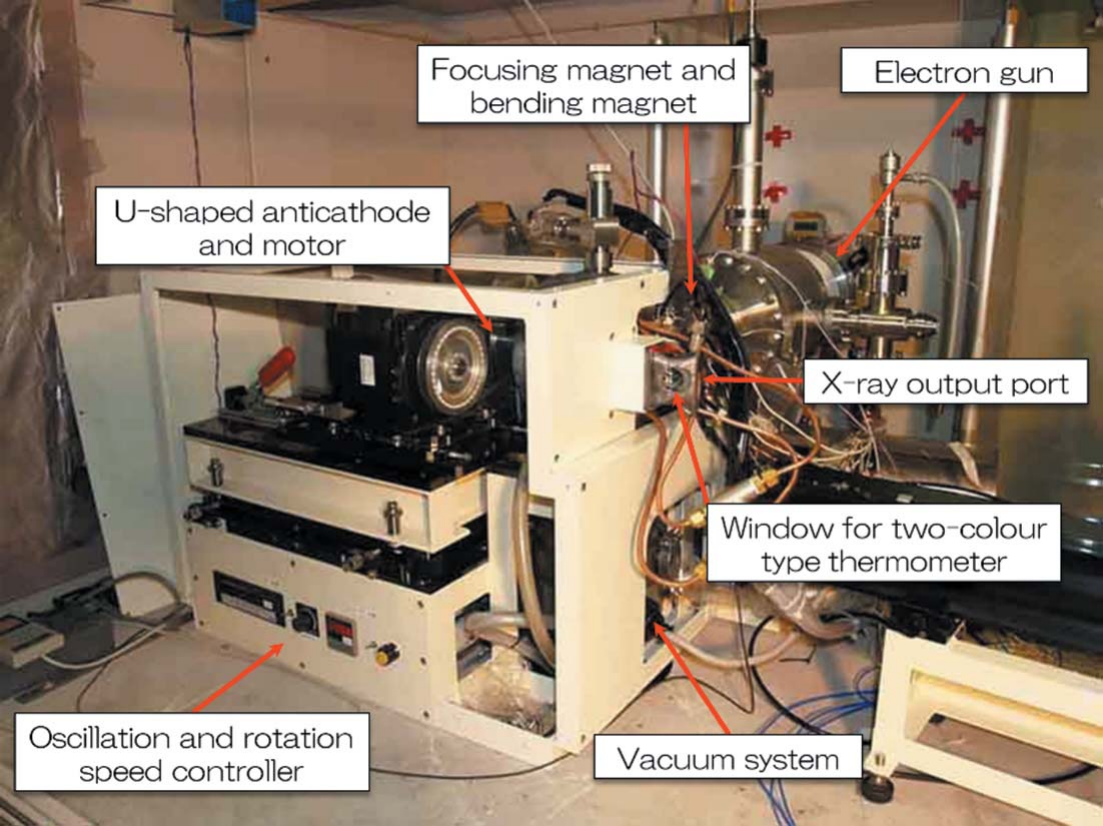
Photograph of the new X-ray generator under test with the U-shaped rotating anticathode. Electrons come from the back on the right and, after being bent horizontally by 180°, irradiate the U-shaped rotating anticathode. X-rays come out in the rightward direction.

**Table 1:** Specifications of the new X-ray generator

Maximum power	120 kV, 75 mA
Target metal	100 mm-diameter Cu
Rotation frequency	100 s^−1^
Cathode material	LaB_6_
Cathode type	Thermionic
Cathode diameter	2 mm
Cathode grid type	Aperture grid
Cathode grid voltage	3 kV
